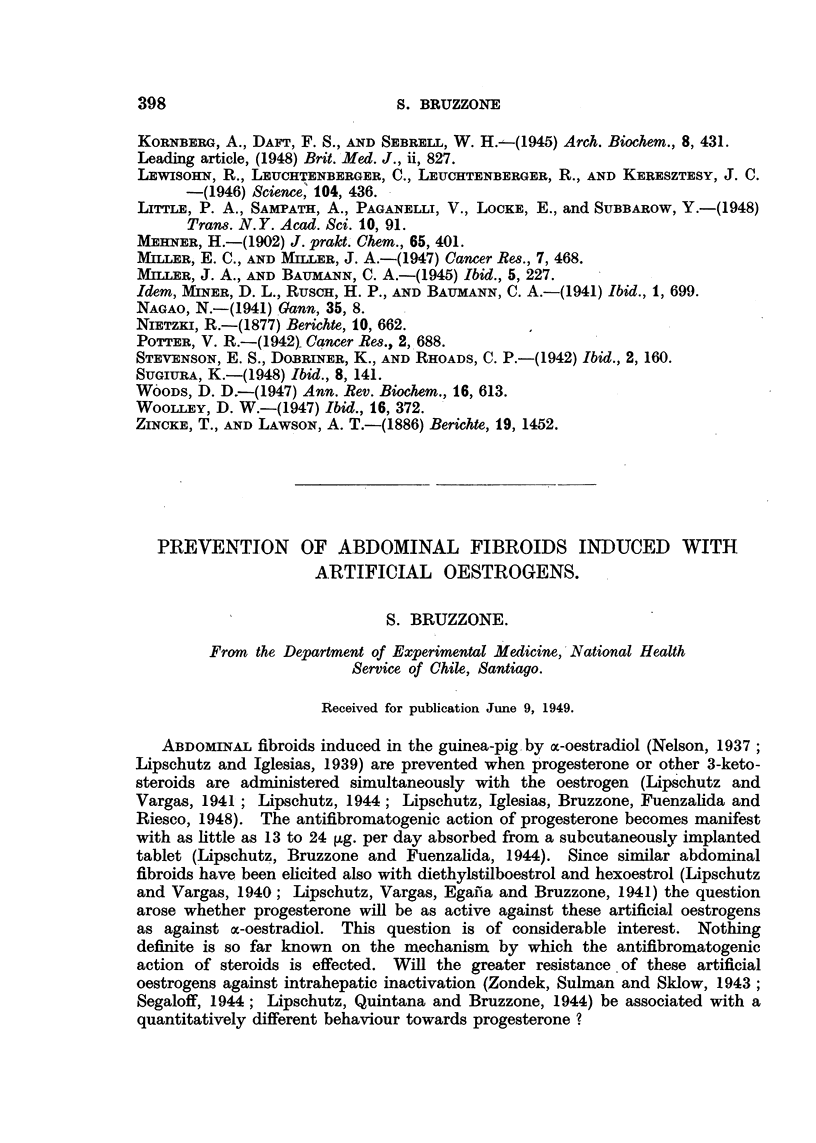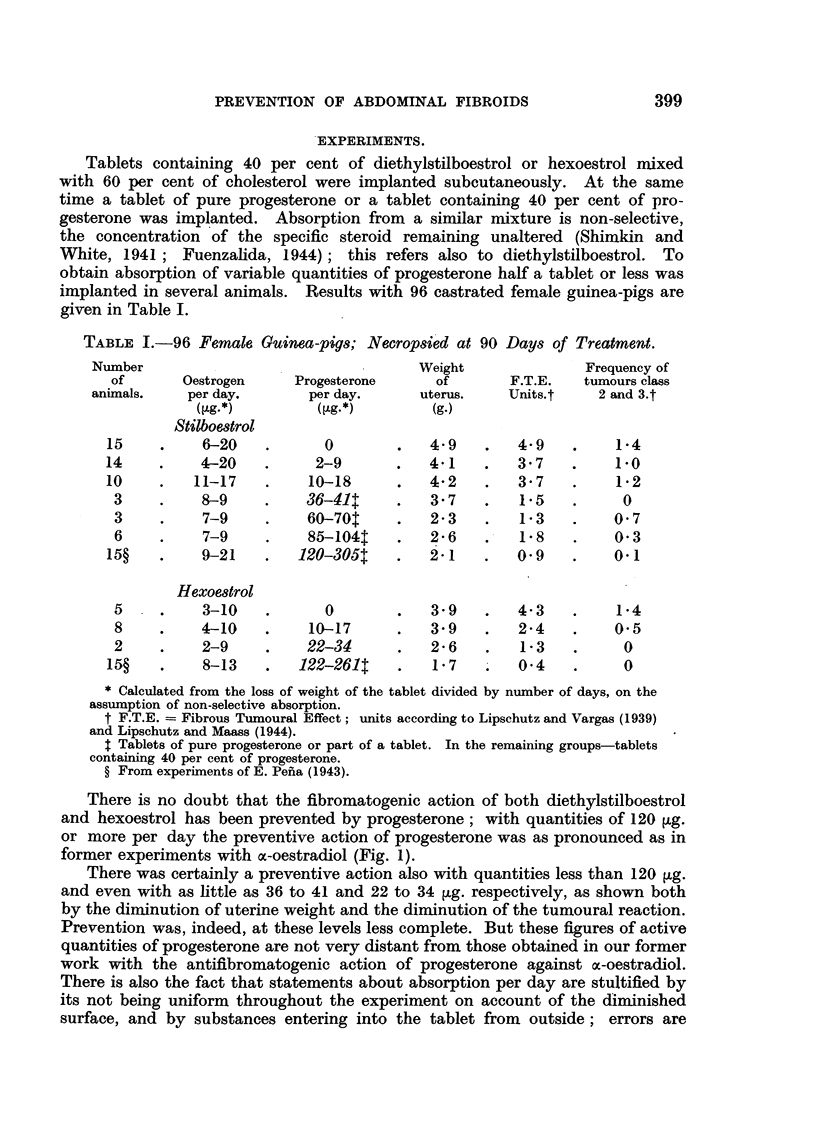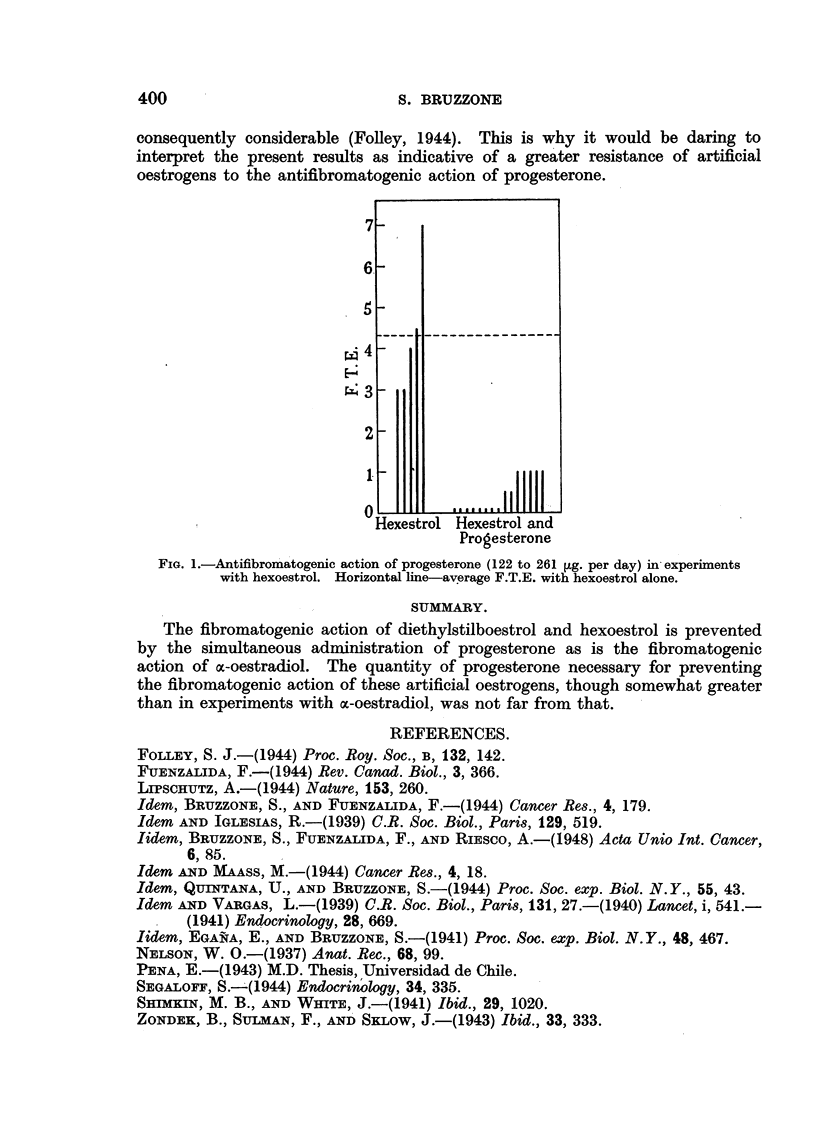# Prevention of Abdominal Fibroids Induced with Artificial Oestrogens

**DOI:** 10.1038/bjc.1949.44

**Published:** 1949-09

**Authors:** S. Bruzzone


### PREVENTION OF ABDOMINAL FIBROIDS INDUCED WITH ARTIFICIAL OESTROGENS.

S. BRUZZONE.

*From the Department of Experimental Medicine, National Health Service of Chile, Santiago.*

Received for publication June 9, 1949.

Abdominal fibroids induced in the guinea-pig by α-oestradiol (Nelson, 1937 ; Lipschutz and Iglesias, 1939) are prevented when progesterone or other 3-keto-steroids are administered simultaneously with the oestrogen (Lipschutz and Vargas, 1941 ; Lipschutz, 1944 ; Lipschutz, Iglesias, Bruzzone, Fuenzalida and Riesco, 1948). The antifibromatogenic action of progesterone becomes manifest with as little as 13 to 24 μg. per day absorbed from a subcutaneously implanted tablet (Lipschutz, Bruzzone and Fuenzalida, 1944). Since similar abdominal fibroids have been elicited also with diethylstilboestrol and hexoestrol (Lipschutz and Vargas, 1940 ; Lipschutz, Vargas, Egaña and Bruzzone, 1941) the question arose whether progesterone will be as active against these artificial oestrogens as against α-oestradiol. This question is of considerable interest. Nothing definite is so far known on the mechanism by which the antifibromatogenic action of steroids is effected. Will the greater resistance of these artificial oestrogens against intrahepatic inactivation (Zondek, Sulman and Sklow, 1943 ; Segaloff, 1944 ; Lipschutz, Quintana and Bruzzone, 1944) be associated with a quantitatively different behaviour towards progesterone ?

EXPERIMENTS.

Tablets containing 40 per cent of diethylstilboestrol or hexoestrol mixed with 60 per cent of cholesterol were implanted subcutaneously. At the same time a tablet of pure progesterone or a tablet containing 40 per cent of progesterone was implanted. Absorption from a similar mixture is non-selective, the concentration of the specific steroid remaining unaltered (Shimkin and White, 1941; Fuenzalida, 1944); this refers also to diethylstilboestrol. To obtain absorption of variable quantities of progesterone half a tablet or less was implanted in several animals. Results with 96 castrated female guinea-pigs are given in Table I.

TABLE I.—96 *Female Guinea-pigs; Necropsied at* 90 *Days of Treatment.*

| Number of animals. | Oestrogen per day. (μg.*) | Progesterone per day. (μg.*) | Weight of uterus. (g.) | F.T.E. Units.† | Frequency of tumours class 2 and 3.† |
|---|---|---|---|---|---|
| | *Stilboestrol* | | | | |
| 15 | 6–20 | 0 | 4·9 | 4·9 | 1·4 |
| 14 | 4–20 | 2–9 | 4·1 | 3·7 | 1·0 |
| 10 | 11–17 | 10–18 | 4·2 | 3·7 | 1·2 |
| 3 | 8–9 | *36–41*‡ | 3·7 | 1·5 | 0 |
| 3 | 7–9 | 60–70‡ | 2·3 | 1·3 | 0·7 |
| 6 | 7–9 | 85–104‡ | 2·6 | 1·8 | 0·3 |
| 15§ | 9–21 | *120–305*‡ | 2·1 | 0·9 | 0·1 |
| | *Hexoestrol* | | | | |
| 5 | 3–10 | 0 | 3·9 | 4·3 | 1·4 |
| 8 | 4–10 | 10–17 | 3·9 | 2·4 | 0·5 |
| 2 | 2–9 | *22–34* | 2·6 | 1·3 | 0 |
| 15§ | 8–13 | *122–261*‡ | 1·7 | 0·4 | 0 |

* Calculated from the loss of weight of the tablet divided by number of days, on the assumption of non-selective absorption.

† F.T.E. = Fibrous Tumoural Effect; units according to Lipschutz and Vargas (1939) and Lipschutz and Maass (1944).

‡ Tablets of pure progesterone or part of a tablet. In the remaining groups—tablets containing 40 per cent of progesterone.

§ From experiments of E. Peña (1943).

There is no doubt that the fibromatogenic action of both diethylstilboestrol and hexoestrol has been prevented by progesterone; with quantities of 120 μg. or more per day the preventive action of progesterone was as pronounced as in former experiments with α-oestradiol (Fig. 1).

There was certainly a preventive action also with quantities less than 120 μg. and even with as little as 36 to 41 and 22 to 34 μg. respectively, as shown both by the diminution of uterine weight and the diminution of the tumoural reaction. Prevention was, indeed, at these levels less complete. But these figures of active quantities of progesterone are not very distant from those obtained in our former work with the antifibromatogenic action of progesterone against α-oestradiol. There is also the fact that statements about absorption per day are stultified by its not being uniform throughout the experiment on account of the diminished surface, and by substances entering into the tablet from outside; errors are consequently considerable (Folley, 1944). This is why it would be daring to interpret the present results as indicative of a greater resistance of artificial oestrogens to the antifibromatogenic action of progesterone.

FIG. 1.—Antifibromatogenic action of progesterone (122 to 261 μg. per day) in experiments with hexoestrol. Horizontal line—average F.T.E. with hexoestrol alone.

SUMMARY.

The fibromatogenic action of diethylstilboestrol and hexoestrol is prevented by the simultaneous administration of progesterone as is the fibromatogenic action of α-oestradiol. The quantity of progesterone necessary for preventing the fibromatogenic action of these artificial oestrogens, though somewhat greater than in experiments with α-oestradiol, was not far from that.

### REFERENCES.

FOLLEY, S. J.—(1944) *Proc. Roy. Soc.*, B, **132**, 142.
FUENZALIDA, F.—(1944) *Rev. Canad. Biol.*, **3**, 366.
LIPSCHUTZ, A.—(1944) *Nature*, **153**, 260.
*Idem*, BRUZZONE, S., AND FUENZALIDA, F.—(1944) *Cancer Res.*, **4**, 179.
*Idem* AND IGLESIAS, R.—(1939) *C.R. Soc. Biol.*, *Paris*, **129**, 519.
*Iidem*, BRUZZONE, S., FUENZALIDA, F., AND RIESCO, A.—(1948) *Acta Unio Int. Cancer*, **6**, 85.
*Idem* AND MAASS, M.—(1944) *Cancer Res.*, **4**, 18.
*Idem*, QUINTANA, U., AND BRUZZONE, S.—(1944) *Proc. Soc. exp. Biol. N.Y.*, **55**, 43.
*Idem* AND VARGAS, L.—(1939) *C.R. Soc. Biol.*, *Paris*, **131**, 27.—(1940) *Lancet*, i, 541.—(1941) *Endocrinology*, **28**, 669.
*Iidem*, EGAÑA, E., AND BRUZZONE, S.—(1941) *Proc. Soc. exp. Biol. N.Y.*, **48**, 467.
NELSON, W. O.—(1937) *Anat. Rec.*, **68**, 99.
PENA, E.—(1943) M.D. Thesis, Universidad de Chile.
SEGALOFF, S.—(1944) *Endocrinology*, **34**, 335.
SHIMKIN, M. B., AND WHITE, J.—(1941) *Ibid.*, **29**, 1020.
ZONDEK, B., SULMAN, F., AND SKLOW, J.—(1943) *Ibid.*, **33**, 333.